# Predicting Long-Term Mortality in TAVI Patients Using Machine Learning Techniques

**DOI:** 10.3390/jcdd8040044

**Published:** 2021-04-16

**Authors:** Marco Penso, Mauro Pepi, Laura Fusini, Manuela Muratori, Claudia Cefalù, Valentina Mantegazza, Paola Gripari, Sarah Ghulam Ali, Franco Fabbiocchi, Antonio L. Bartorelli, Enrico G. Caiani, Gloria Tamborini

**Affiliations:** 1 Centro Cardiologico Monzino, IRCCS, 20138 Milan, Italy; Mauro.Pepi@ccfm.it (M.P.); Laura.Fusini@ccfm.it (L.F.); Manuela.Muratori@ccfm.it (M.M.); Claudia.Cefalu@ccfm.it (C.C.); Valentina.Mantegazza@ccfm.it (V.M.); Paola.Gripari@ccfm.it (P.G.); Sarah.Ghulamali@ccfm.it (S.G.A.); Franco.Fabbiocchi@ccfm.it (F.F.); Antonio.Bartorelli@ccfm.it (A.L.B.); Gloria.Tamborini@ccfm.it (G.T.); 2 Department of Biomedical and Clinical Sciences “Luigi Sacco”, University of Milan, 20157 Milan, Italy; 3 Department of Electronics, Information and Biomedical Engineering, Politecnico di Milano, 20133 Milan, Italy; enrico.caiani@polimi.it

**Keywords:** machine learning, TAVI, mortality prediction, aortic valve disease

## Abstract

Background: Whereas transcatheter aortic valve implantation (TAVI) has become the gold standard for aortic valve stenosis treatment in high-risk patients, it has recently been extended to include intermediate risk patients. However, the mortality rate at 5 years is still elevated. The aim of the present study was to develop a novel machine learning (ML) approach able to identify the best predictors of 5-year mortality after TAVI among several clinical and echocardiographic variables, which may improve the long-term prognosis. Methods: We retrospectively enrolled 471 patients undergoing TAVI. More than 80 pre-TAVI variables were collected and analyzed through different feature selection processes, which allowed for the identification of several variables with the highest predictive value of mortality. Different ML models were compared. Results: Multilayer perceptron resulted in the best performance in predicting mortality at 5 years after TAVI, with an area under the curve, positive predictive value, and sensitivity of 0.79, 0.73, and 0.71, respectively. Conclusions: We presented an ML approach for the assessment of risk factors for long-term mortality after TAVI to improve clinical prognosis. Fourteen potential predictors were identified with the organic mitral regurgitation (myxomatous or calcific degeneration of the leaflets and/or annulus) which showed the highest impact on 5 years mortality.

## 1. Introduction

Since its introduction in 2002, transcatheter aortic valve implantation (TAVI) has evolved dramatically due to its advantage treating patients with symptomatic severe aortic valve stenosis (AS) at high or prohibitive risk for surgical aortic valve replacement (SAVR). Currently, TAVI is a consolidated procedure and guidelines recommend TAVI to improve symptoms and survival in symptomatic patients at high surgical risk [1]. Recent evidence has also extended TAVI in selected intermediate risk patients [1,2], and even low-risk candidates might be offered TAVI in the near future [3]. At 5 years, no difference in mortality between TAVI and SAVR for high-risk patients has been observed [3]. More recently, it was demonstrated that 5-year mortality rates of TAVI and SAVR were not statistically different in a population at intermediate surgical risk, although incidence of death was higher in a subset of patients undergoing transapical TAVI [4]. Despite TAVI having become the gold standard treatment for high-risk patients with severe symptomatic AS, demonstrating results either superior or at least non-inferior to SAVR, the reported all-cause mortality rate in high-risk patients ranges from 6.7% to 14.5% at 1 year after TAVI and grows up to about 47% at 5 years [5,6]. While SAVR mortality is mainly due to well-known parameters and factors related to mechanical or biological disfunction over time, TAVI long-term mortality prediction is still unknown. Therefore, the evaluation of mortality predictors in long-term follow-up after TAVI is of utmost importance for patient selection, risk stratification, tailoring therapy, and correctly informing the patient about long-term prognosis after the procedure.

Machine learning (ML) solutions have emerged as highly effective methods for prediction and decision-making, allowing more accurate prognoses by modeling linear and nonlinear interactions among many variables [7]. ML showed promising results in different medical fields [8,9] and were recently applied to predict in-hospital [10] or 1-year mortality after TAVI [11]. We hypothesized that learning algorithms may allow predictive features undetected by conventional statistical methods to be discovered in order to improve risk definition and prognosis after TAVI procedure. We therefore aimed to develop a novel risk prediction approach based on an ML model able to predict the mortality rate at 5-year follow-up (5FU) after TAVI.

## 2. Materials and Methods

### 2.1. Study Population

Patients affected by symptomatic severe AS, as defined by guidelines [1,2], who underwent TAVI at Centro Cardiologico Monzino IRCCS (Milan, Italy) between 2008 and 2014 were included. Patients were considered as high or intermediate operative risk for conventional SAVR by a multidisciplinary heart team. TAVI procedures were performed using a balloon-expandable SAPIEN or SAPIEN XT prosthesis (Edwards Lifesciences, Irvine, CA, USA), which was delivered through either the transfemoral or the transapical approach. Both valves were available in 23-, 26-, 29-, and 31-mm sizes. Prosthesis sizing was based on aortic annulus measurements using 3-dimensional imaging techniques (multidetector row computed tomography or transesophageal echocardiography). Baseline patient data including echocardiographic data, laboratory results, diagnosis, and clinical status/symptoms were retrospectively analyzed. Patients were followed up until death. The study population was allocated into 2 groups: Patients who were living at 5 years from the TAVI (survivor) and patients who died at 5 years after TAVI (non-survivor). Survival and causes of death were assessed for all patients by consulting the patient’s medical files. All-cause of mortality at 5 years after TAVI was the main end-point. The study was approved by the local ethical committee and all enrolled patients signed informed consent.

### 2.2. Clinical Variables

For each patient, 83 pre-TAVI variables were considered. All variables, as well as the descriptive statistics, can be found in Table S1 in the Supplementary Material. Baseline transthoracic echocardiography, including M-mode, and 2D and Doppler evaluation, was performed using commercially available ultrasound system (Vivid 7 and E9, GE Medical Systems, Horten, Norway; and iE33, Philips Medical Systems, Andover, MA, USA). Left ventricular (LV) assessment was performed as recommended, including linear dimensions at parasternal long-axis view and mass evaluation [12]. LV volumes and LV ejection fraction were calculated in accordance with the Simpson’s method, as well as and the left atrial volume. Severity of mitral and tricuspid valve regurgitation (MR, TR) was assessed in accordance with the guidelines [13]. Functional MR was defined as no or minor pathology of the mitral valve leaflets, annulus, and chordae associated with dilated LV with global or regional wall motion abnormalities. Organic MR was defined as myxomatous or calcific degeneration of the leaflets and/or annulus [14]. Aortic valve area was derived from the continuity equation according to guidelines [15]. The mean trans-aortic valve gradient was measured on continuous wave Doppler acquisitions using either the apical 5- or 3-chamber view and the right parasternal view [15]. Aortic annulus area was estimated with the assumption of circular configuration, and the prosthesis-to-indexed annulus size ratio was derived. Maximal TR jet velocity combined with inferior vena cava respiratory variation was used to calculate systolic pulmonary arterial pressure [16]. Baseline patient data were used to calculate Cardiac Operative Risk Evaluation II (EuroSCORE II) [17], which was considered as a handcrafted feature [18]. Parameters were defined according to the definitions applied in the EuroSCORE II. Additional baseline characteristics, potentially relevant to mortality evaluation, were also collected, such as hemoglobin, C-reactive protein, serum albumin, aspartate transaminase, alanine aminotransferase, and total bilirubin. Typical symptoms of aortic stenosis (angina, dyspnea, and syncope) were recorded if mentioned in the clinical history. Porcelain aorta and hostile chest were noted according to recent definitions [19]. The procedure was considered as urgent if patients required intervention on current admission for medical reasons.

### 2.3. Study Design

**Figure 1:**
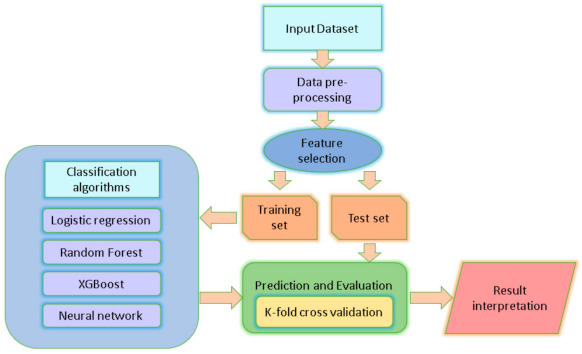
Computational methods. Schematic workflow for the construction of classification models including feature selection, cross-validation to evaluate the discriminant performance, and resulting interpretation.

This study retrospectively evaluated three widely used supervised classification ML algorithms using different classifiers to predict the occurrence of all-cause of death at 5-year mortality after TAVI: Random forest (RF), extreme gradient boosting (XGBoost), and multilayer perceptron (MLP) [20,21]. In addition, a logistic regression (LR) model was implemented. We derived the LR model using a multivariate analysis. Models were constructed in Python version 3.7 (Python Software Foundation) using the scikit-learn and keras packages. Figure 1 shows the analysis workflow schematically.

RF and XGBoost are tree-based ML algorithms, developed to improve tree-based ensemble’s performance, while not increasing the bias significantly. The Bootstrap aggregating technique was used in RF to build independent trees, where each tree is trained on a sample drawn from the training set, which makes the model an effective learner for smaller datasets. XGboost is an improved algorithm based on the gradient-boosting method to fit an ensemble of weak learners trained sequentially such that each one of them is encouraged to correct mistakes of previous learners, which increases the accuracy and prevents overfitting. Sequentially combining decision trees as base learners in a way that each learner fits to the residuals from the precious step has the advantage of accelerating the learning process.

MLP is a neuron-based model for nonlinear function approximation, with a number of neural units through several layers. With a minimum of three layers (i.e., the input, hidden, and output layers), the network changes its weight in proportional to the error between the true and predicted output by backpropagation algorithm, the standard algorithm for the supervised-learning process.

Before proceeding with the analysis, the dataset underwent preprocessing for data optimization and consistency. There were 83 variables in the initial dataset. Different approaches were adopted to remove non-informative or redundant variables including dropping 0-variance features and highly correlated variables (Table S2, Supplementary Materials). A total of 70 predictors remained in the dataset. As a requisite for many ML techniques and feature selection methods, Z-score standardization was applied for continuous predictors and dummy coding and target coding [22] for nominal and categorical variables, respectively.

Considering the large number of available variables, different feature selection methods were evaluated. Feature selection is defined as the process of reducing the number of input variables needed to predict the target variable, removing non-informative or redundant predictors that might add uncertainty, thus degrading the performance of the model [23]. For each algorithm, feature selection was performed using least absolute shrinkage and selection operator (LASSO), gradient-boosting machine (GBM), Boruta, and RF [24,25,26]. In addition, recursive feature elimination (RFE) was applied to the best performing model.

In order to train algorithms and assess their performance and general error estimation, a stratified ten-fold cross-validation was implemented; thus, the dataset was cyclically split into ten equally sized folds, preserving the percentage of samples for each class (i.e., survivor and non-survivor at 5 years after TAVI), in which nine folds were used to train the model (90% of the cohort) and one to validate model performance (10% of data). This method maximized the use of data for both training and testing, reducing the variance in prediction error for an accurate estimate of model prediction performance.

In each training set, to optimize the ML model’s hyperparameters, an iterative strategy with different combinations of parameters and five-fold cross-validation was performed. Further details on the model’s hyperparameters are presented in Table S3 in the Supplementary Materials.

### 2.4. Model Evaluation

ML performances on the testing set were evaluated by using the area under the receiver-operating curve (AUC). Moreover, for the best resulting AUC model, additional metrics were computed, such as accuracy, sensitivity, positive predictive value (PPV), and F1-score, and a comparison with the EuroSCORE II, which represents the most used score in TAVI, was reported.

To determine the major relevant predictors of the study outcome for the best ML model, the permutation feature importance (PFI) approach was measured [27]. PFI is an algorithm for measuring the association of individual variables with model accuracy, where variables’ values are iteratively permutated within the test set, and the prediction error of the model is measured. A variable is considered important if permuting its value decreases the model’s discriminative capability, as the model relies heavily on that variable. The F1-score was recalculated with permutated data to determine variable importance.

For ML model interpretability, an additive feature attribution method (Shapley additive explanations) was proposed [28], which defines a weighted linear regression by using data and predictions of the analyzed model to point out the positive or negative relationship of feature value on the prediction. Results were discussed with expert medical cardiologists, and clinical explanations were reported.

### 2.5. Statistical Analysis

Continuous data are presented as mean ± standard deviations or median (twenty-fifth–seventy-fifth percentile) as appropriate, and categorical variables as frequencies (%). Differences between survived and not-survived patients were assessed using an unpaired Student’s *t*-test for continuous variables (and the Welch’s corrected version, as appropriate) or the Mann–Whitney U test, whilst an χ^2^ test was applied for categorical data. The DeLong test was used to measure the difference between AUC. Significant variables at univariate analysis were included in the multivariate LR model for the identification of independent predictors. Statistical analyses were conducted with SPSS 26 (SPSS Inc., Chicago, IL, USA), and values of *p* < 0.05 were considered statistically significant.

## 3. Results

Of the 475 patients with severe AS undergoing successful TAVI, 4 patients were excluded for incomplete data. The final population included 471 patients, who were divided into 2 groups according to whether the patients survived or died during the 5 years after TAVI; 259 (55%) were in the survivor group (mean age 80 ± 6 years, 36.7% men), and 212 (45%) were in the non-survivor group (mean age 82 ± 6 years, 35.8% men). Specifically, 12 patients (2%) died from stroke and cardiovascular death occurred in 93 patients (20%). According to EuroSCORE II, patients were at high and intermediate surgical risk in 75% and 25%, respectively. Table 1 reports the baseline characteristics of the study population, which had a prevalence of females (63.7%) and a mean age of 81 years. The majority of the patients presented hypertension (87.3%), dyspnea (91.7%), and coronary artery disease (57.3%). Clinical and echocardiographic parameters of the study cohort dichotomized based on 5 years mortality status are presented in Table S1 in the Supplementary Materials.

**Figure 2:**
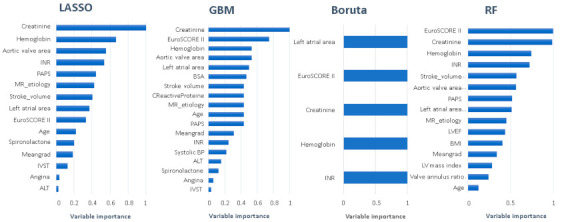
Feature selection methods. The most relevant variables identified for each method. MR, mitral regurgitation; ALT, alanine aminotransferase; IVST, interventricular septal thickness; Meangrad, mean aortic pressure gradient; INR, international normalized ratio; PAPS, pulmonary artery systolic pressure; BSA, body surface area; BMI, body mass index; LV, left ventricular; EF, ejection fraction.

Figure 2 shows the results of the feature selection analysis: Using LASSO, 15 potential predictors were selected for the ML analysis, with GBM, 18 predictors were identified, while Boruta and RF respectively identified 5 and 15 predictors. Creatinine and hemoglobin were shared across all methods.

**Figure 3:**
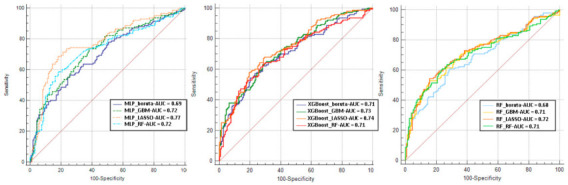
Evaluation of mortality prediction for different machine learning (ML) models. Receiver operating characteristic curve from ten-fold cross-validation for mortality prediction. AUC, area under the curve; MLP, multilayer perceptron; GBM, gradient boosting machine; XGBoost, extreme gradient boosting; RF, random forest; LASSO, least absolute shrinkage and selection operator.

Algorithm discrimination of tenfold cross-validation is presented for each ML model in Figure 3. The best AUC was reached combining LASSO as the feature selection method and MLP as the model, which was able to predict the outcome with good performance (AUC: 0.77; 95% confidence interval (CI): 0.73 to 0.81) with significant difference in AUC compared with MLP + GBM (AUC: 0.72; 95% CI: 0.68 to 0.76), MLP + BORUTA (AUC: 0.69; 95% CI: 0.65 to 0.73), MLP + RF (AUC: 0.72; 95% CI: 0.67 to 0.75), XGBoost + GBM (AUC: 0.73; 95% CI: 0.69 to 0.77), XGBoost + BORUTA (AUC: 0.71; 95% CI: 0.65 to 0.75), XGBoost + RF (AUC: 0.71; 95% CI: 0.65 to 0.76), RF + LASSO (AUC: 0.72; 95% CI: 0.68 to 0.76), RF + GBM (AUC: 0.71; 95% CI: 0.66 to 0.76), RF + BORUTA (AUC: 0.68; 95% CI: 0.63 to 0.73), and RF + RF (AUC: 0.71; 95% CI: 0.66 to 0.76), while there was no significant difference versus XGBoost + LASSO (AUC: 0.74; 95% CI: 0.71 to 0.77).

Table 2 reports the variables included in the LR model. At multivariate analysis only the mean aortic pressure gradient, organic etiology of MR, creatinine, and hemoglobin were the independent predictors associated with 5-year mortality after TAVI.

**Figure 4:**
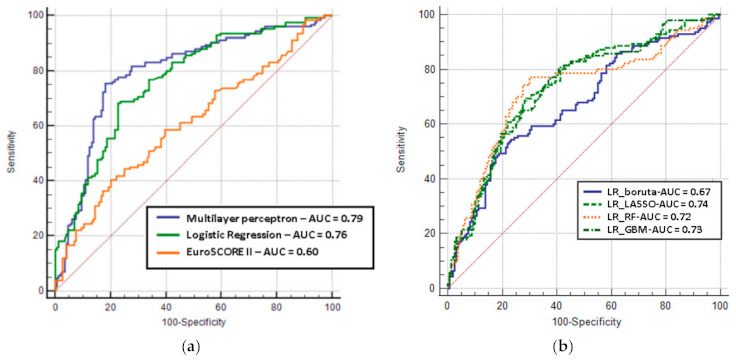
Receiver operating characteristic curves for prediction of 5-year mortality: (**a**) multilayer perceptron vs. logistic regression vs. EuroSCORE II, (**b**) logistic regression models using different feature selection methods. AUC, area under the curve; LR, logistic regression; GBM, gradient boosting machine; RF, random forest; LASSO, least absolute shrinkage and selection operator.

After RFE, LASSO + MLP had the best discrimination compared to multivariate LR and EuroSCORE II (MLP: 0.79; 95% CI: 0.75 to 0.83 vs. LR: 0.76; 95% CI: 0.73 to 0.79 vs. EuroSCORE II: 0.60; 95% CI: 0.55 to 0.62), although no significant difference was observed between MLP and multivariate LR (Figure 4a). Considering the different feature selection methods, there was no performance improvement in LR (Figure 4b): LR + BORUTA (AUC: 0.67; 95% CI: 0.64 to 0.71), LR + LASSO (AUC: 0.74; 95% CI: 0.69 to 0.78), LR + RF (AUC: 0.72; 95% CI: 0.68 to 0.76), and LR + GBM (AUC: 0.73; 95% CI: 0.69 to 0.77).

RFE identified 14 pre-treatment variables as the most relevant predictors of mortality in TAVI patient at 5-years follow-up: MR etiology, stroke volume index, interventricular septal thickness, left atrium area, aortic valve area, mean aortic pressure gradient, creatinine, alanine aminotransferase, hemoglobin, international normalized ratio, age, spironolactone, angina, and EuroSCORE II (Table 3). Specifically, compared with the survivor group, the non-survivor group had a higher age (mean 82 ± 6 years vs. 80 ± 6 years; *p* = 0.025), higher creatinine (median 1.16 (0.91–1.48) mg/dL vs. 0.92 (0.77–1.20) mg/dL; *p* < 0.001), lower hemoglobin (mean 11.9 ± 1.6 g/dL vs. 12.4 ± 1.7 g/dL; *p* < 0.001), lower mean aortic pressure gradient (mean 48 ± 15 mmHg vs. 53 ± 14 mmHg; *p* < 0.001), higher left atrium area (mean 28 ± 7 cm^2^ vs. 26 ± 6 cm^2^; *p* < 0.001), and higher aortic valve area (mean 0.66 ± 0.14 cm^2^ vs. 0.64 ± 0.14 cm^2^; *p* = 0.078). In addition, higher prevalence of organic MR was found in the non-survivor group compared to the survivor group (46.7% vs. 29.7%; *p* < 0.001).

The PPV of the MLP for predicting mortality after TAVI was 0.73, the sensitivity was 0.71, and the F1-score was 0.71. The overall accuracy of the MLP was 0.73 (Table 4). Codes used for MLP development are made publicly available in the Supplementary Materials.

**Figure 5:**
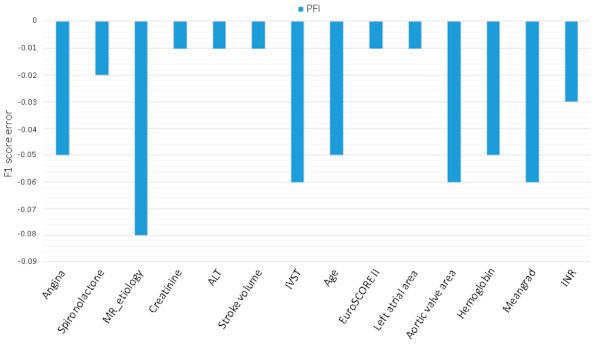
Permutation feature importance permutation feature importance (PFI) method. More relevant features are associated with more negative values. MR, mitral regurgitation; ALT, alanine aminotransferase; IVST, interventricular septal thickness; Meangrad, mean aortic pressure gradient; INR, international normalized ratio.

**Figure 6:**
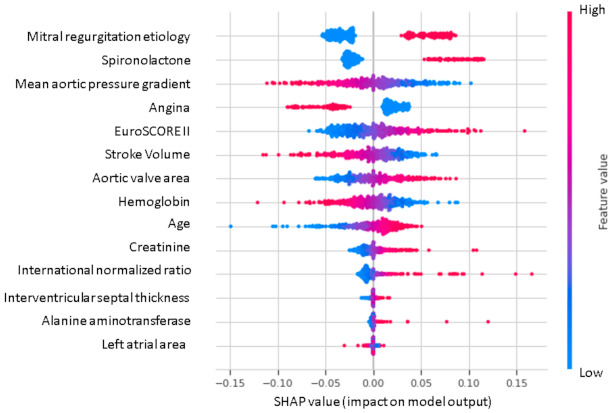
Shapley additive explanations value plot. The horizontal axis shows whether the effect of the feature is associated with a higher or lower prediction, while the color indicates whether the value of the feature is high (red) or low (blue) for a given observation.

Assessing PFI (Figure 5) identified features important to model accuracy for mortality prediction after TAVI, with organic MR showing the highest impact regarding 5-year mortality, followed by the mean aortic pressure gradient. In Figure 6, the effect of each features on the ML classifier.

## 4. Discussion

In this retrospective study, we presented a novel ML approach for the prediction of 5-year mortality after TAVI. To the best of our knowledge, no research has been conducted using ML in reporting longitudinal data in the long-term after TAVI. The main results were the following: (i) MLP model achieved the best AUC (0.79) in predict mortality at 5 years after TAVI; (ii) novel features, never considered in previous mortality risk scores in TAVI patients, were identified.

The assessment of risk factors for long-term mortality after TAVI is crucial to improve clinical decision-making and prognosis. In this context, ML may represent a valid computational tool able to manage a high number of variables and interactions among them, thus integrating the multitude of predictors, which represents a challenge for the clinician. ML-based prognostic tools often discover unexpected variables and interactions, allowing the recognition of potentially new predictors [29]. Lopes et al. [11] achieved the highest AUC (0.70) with a random forest classifier in predicting 1-year mortality after TAVI, while for in-hospital mortality after TAVI, LR was the best model (AUC: 0.92) [10]. Based on our results, ML models might have an important clinical role in evaluating the long-term mortality risk after TAVI, incorporating a multitude of information to accurately represent the clinical scenario under investigation. In the future, this might allow a better evaluation of different treatment options and improve patients’ selection, especially considering intermediate- and low-risk patients. In this analysis, only pre-TAVI echocardiographic and clinical variables were considered. It is reasonable to hypothesize that the longer the follow-up, the greater the need to also include post-TAVI variables to better tune the model and make the prediction more robust and updated over time. However, the inclusion of intraoperative or post-treatment variables was beyond our scope of aiding treatment decision. With expanding indications for TAVI, our findings may support clinicians in assessing prognosis after TAVI, which is paramount for accurate patient information regarding the outcome of the procedure.

Among ML models, MLP showed slightly better predictive abilities. Our findings did not show significant differences in AUC between MLP and LR to estimate 5FU mortality. There are two possible hypotheses for this: (1) Complex non-linear relationships do not exist, at least among the selected predictors; and (2) the size of the study cohort might limit the model’s optimization. Nevertheless, as clinicians continue to gather significant amounts of patient data, the role of ML in medicine is expected to increase, becoming an essential tool for clinicians in different clinical contexts, including decision-making, diagnosis and event prediction. Different from conventional statistical models, ML models are capable of capturing more complex non-linear relationships between data, with potential benefits in terms of mortality prediction. Furthermore, unexpected predictor variables, which non-linearly interact with stronger predictors, could improve clinical decision-making, supporting diagnosis and therapy planning. Moreover, the incorporation of new data during the training procedure could further improve the ML model performance over time. Finally, using the ML approach, several variables usually excluded from the analysis based on traditional statistics, as a consequence of their inherent methodological limitations, were included in the same examination. However, besides the power in identifying complex patterns and in providing high prediction accuracy, many ML models lack transparency, which refers to an understanding of how the model works and what the model actually computes, thus preventing the direct identification and evaluation of the relationships between the input variables.

To try to cope with this limitation, we conducted a posteriori analysis to understand which features were more relevant in the achievement of the results. The PFI method identified organic MR as a strong predictor of mortality. In addition, age, aortic valve area, mean aortic pressure gradient, and hemoglobin levels proved to be relevant predictors for mortality prediction, playing an important role in this context. As a result, these variables combined altogether assumed a more relevant importance in the definition of 5-year mortality risk after TAVI. Other variables, such as spironolactone, international normalized ratio, and creatinine, also appeared to be relevant factors in assessing mortality. The variable importance technique PFI provides a global insight into the model’s behavior, considering interactions between features; however, this method does not reflect the intrinsic feature effects on the target variable. Interestingly, the EuroSCORE II resulted in important predictors for the MLP. Although the EuroSCORE’s performance in a long follow-up is limited (Figure 4), its predicting ability was included into the 5-year estimate.

From a clinical point of view, some pre-procedural patient characteristics included in Shapley additive explanations analysis such as anemia, older age, renal dysfunction, high mean aortic gradient, smaller aortic area, and atrial dilatation are not only incorporated in traditional risk scores showing a negative relationship with the outcome after TAVI, but also have a negative prognostic significance in the general population [30,31].The presence of angina is associated with a more favorable prognosis at 5FU, probably because angina onset may facilitate an earlier diagnosis of severe AS in comparison with patients without angina, who may afterwards develop heart failure symptoms, which are associated with a worst prognosis. Regarding MR etiology as a negative survival prognostic factor in TAVI patients, a significant association has been demonstrated between a 3-year mortality rate and pre-TAVI organic MR [32]. In fact, while both functional and organic moderate/severe pre-TAVI MR was associated with a higher mortality rate at 1-year follow-up, a significant improvement in regurgitation severity was observed mainly in patients with functional MR, and the persistence of significant regurgitation in organic cases had a negative impact on 3-year mortality [32]. Finally, another novelty of our study is that low stroke volume (SV) was associated with higher mortality. A low SV is generally due to LV dysfunction and an increased mortality risk in classical low flow-low gradient AS has been largely proved [33]. However, low SV is also frequently described in patients affected by paradoxical low-flow low-gradient AS with small LV volumes and preserved LV ejection fraction. Low SV is known to have an important negative impact on survival of these patients when not undergoing surgery; however, controversial data exist on clinical outcomes after surgery or TAVI [34,35].

### Limitations

The present study has some limitations. First, the size of the dataset was limited which may affect the model’s performance. Second, it was a single-center study. The inclusion of datasets from multiple centers would provide more information about the generalization of the model. Third, the dataset included patients undergoing TAVI until 2014, thus including only patients at high and intermediate risk; therefore, we may not extrapolate our results to lower risk cases. Furthermore, additional variables may impact the model’s outcome, such as natriuretic peptides and troponin, or computed tomography parameters. Specifically, morphological features could have improved the model discrimination. Recently, statistical shape models have attracted much attention as methods to improve the robustness and accuracy of feature extraction. These methods, in the context of the heart valve’s morphology analysis, could be used for capturing features of the global shape of the valve, rather than reducing it to conventional geometric measurements [36]. In addition, the lack of transparency and the difficult interpretation of the ML model may affect its reliability into clinical practice. Regardless, it is likely that a synergistic relationship between ML and medicine will become more pronounced, due to the rapid improvements of ML algorithms and the increasing digitalization of data.

## 5. Conclusions

Several risk scores have been proposed to predict outcomes after TAVI, but optimizing the selection of patients remains an unmet clinical need. This analysis confirmed that 5-year mortality prediction after TAVI is challenging even using ML techniques. We presented a new approach to long-term mortality prediction in TAVI patients based on different analytic methods and different variables compared with previous risk scores. By using an ML model, several new variables were highlighted as potentially influencing long-term prognosis.

## Figures and Tables

**Table 1:** Baseline characteristic of the population.

Characteristics	n = 471
Age, years	81 ± 6
Female, n (%)	300 (63.7%)
Body mass index, kg/m^2^ Overweight (BMI 25 to <30) Obesity (BMI 30 or higher)	25 ± 5 154 (32.7%) 68 (14.4%)
Hypertension	411 (87.3%)
Diabetes mellitus	122 (2.6%)
Dyslipidemia	276 (58.6%)
Angina	147 (31.2%)
Dyspnea	432 (91.7%)
Syncope	87 (18.5%)
COPD	131 (27.8%)
NYHA functional class III or IV	369 (78.3%)
EuroSCORE II	16 (10–21)
Previous stroke	60 (12.7%)
Porcelain aorta	32 (6.8%)
Cardiac history	
Coronary artery disease	270 (57.3%)
Previous myocardial infarction	92 (19.5%)
Previous PCI	144 (30.6%)
Previous CABG	71 (15.1%)
Atrial fibrillation	86 (18.3%)
Procedural characteristics	
Prosthesis size	
23-mm	195 (41.4%)
26-mm	228 (48.4%)
29-mm	41 (8.7%)
31-mm	7 (1.5%)
Pre-operative echocardiographic characteristics	
LVEDV index (mL/m^2^)	54 (43–69)
LVESV index (mL/m^2^)	21 (16–34)
LVEF (%)	59 (48–66)
LV mass index (g/m^2^)	147 ± 39
Left atrial volume index (mL/m^2^)	57 ± 24
Aortic valve area (cm^2^)	0.65 ± 0.14
Mean aortic pressure gradient (mmHg)	51 ± 15
Peak aortic pressure gradient (mmHg)	82 ± 22
PAPS (mmHg)	42 ± 12
Aortic regurgitation ≥2	120 (25.5%)
Mitral regurgitation ≥2	144 (30.6%)
Tricuspid regurgitation ≥2	89 (18.9%)
MR etiology	
Functional MR	295 (62.6%)
Organic MR	176 (37.4%)

BMI, body mass index; MR, mitral regurgitation; COPD, chronic obstructive pulmonary disease; NYHA, New York Heart Association; PCI, percutaneous coronary intervention; CABG, coronary artery bypass graft; LV, left ventricular; EDV, end diastolic volume; ESV, end systolic volume; EF, ejection fraction; PASP, pulmonary artery systolic pressure.

**Table 2:** Univariate and multivariate regression analysis.

	Univariate		Multivariate	
	OR (95% CI)	*p*-Value	OR (95% CI)	*p*-Value
Age, years	1.035 (1.004–1.066)	0.028	1.031 (0.996–1.067)	0.079
Left ventricular ejection fraction, %	0.975 (0.961–0.990)	0.001	1.004 (0.982–1.025)	0.745
Left atrial area, cm^2^	1.062 (1.030–1.095)	<0.001	1.011 (0.974–1.049)	0.565
Mean aortic pressure gradient, mmHg	0.978 (0.966–0.991)	0.001	0.982 (0.966–0.998)	**0.025**
Mitral regurgitation ≥2	1.773 (1.194–2.633)	0.005	1.129 (0.701–1.818)	0.617
Organic mitral regurgitation	2.071 (1.417–3.026)	<0.001	1.642 (1.071–2.517)	**0.023**
Tricuspid regurgitation ≥2	1.950 (1.221–3.114)	0.005	0.860 (0.465–1.590)	0.631
Pulmonary artery systolic pressure, mmHg	1.031 (1.014–1.048)	<0.001	1.012 (0.990–1.033)	0.284
New York Heart Association ≥3	1.864 (1.177–2.951)	0.008	1.133 (0.661–1.943)	0.649
Diuretics	2.191 (1.410–3.405)	<0.001	1.206 (0.709–2.052)	0.489
Spironolactone	2.185 (1.403–3.401)	0.001	1.607 (0.907–2.664)	0.066
Creatinine, mg/dL	2.819 (1.776–4.473)	<0.001	1.941 (1.257–2.996)	**0.003**
Hemoglobin, g/dL	0.818 (0.732–0.915)	<0.001	0.867 (0.776–0.992)	**0.022**
International normalized ratio	4.735 (1.943–11.539)	0.001	1.992 (0.825–4.811)	0.125
Atrial fibrillation	2.740 (1.682–4.463)	<0.001	1.693 (0.898–3.195)	0.104

Only variables with a univariate *p*-value < 0.05 were allowed to enter the multivariate logistic regression analysis.

**Table 3:** Prediction selected for 5-year mortality prediction after transcatheter aortic valve implantation (TAVI).

	Survivor (n = 259)	Non-Survivor (n = 212)	*p*-Value
	*Echocardiographic parameters*		
Mitral regurgitation etiology, n (%)	Functional 182 (70.3%) Organic 77 (29.7%)	Functional 113 (53.3%) Organic 99 (46.7%)	<0.001
Stroke volume index, mL/m^2^	42 ± 8	40 ± 9	0.020
Interventricular septal thickness, mm	13 ± 2	14 ± 2	0.496
Left atrium area, cm^2^	26 ± 6	28 ± 7	<0.001
Aortic valve area, cm^2^	0.64 ± 0.14	0.66 ± 0.14	0.078
Mean aortic pressure gradient, mmHg	53 ± 14	48 ± 15	0.001
	*Blood chemistry tests*		
Creatinine, mg/dL	0.92 (0.77–1.20)	1.16 (0.91–1.48)	<0.001
Alanine aminotransferase, UI/L	17 (12–23)	16 (12–22)	0.448
Hemoglobin, g/dL	12.4 ± 1.7	11.9 ± 1.6	<0.001
International normalized ratio	1.05 ± 0.19	1.17 ± 0.42	<0.001
	*Other patient characteristics*		
Age, years	80 ± 6	82 ± 6	0.025
Spironolactone, n(%)	42 (16.2%)	63 (29.7%)	<0.001
Angina, n(%)	90 (34.7%)	57 (26.9%)	0.057
EuroSCORE II, %	14 (8–20)	18 (12–25)	<0.001

*p*-Value, survivor vs. non-survivor (unpaired Student’s *t*-test, Mann–Whitney U test, or χ^2^ test).

**Table 4:** Performance metrics of multilayer perceptron model.

Algorithm	Feature Selection Method	AUC	Accuracy	Positive Predictive Value	Sensitivity	F1-Score
multilayer perceptron	LASSO + RFE	0.79	0.73	0.73	0.71	0.71

AUC, area under the receiver operating curve; LASSO, least absolute shrinkage and selection operator; RFE, recursive feature elimination.

## Data Availability

Data cannot be made available.

## Supplementary Materials

The following are available online at https://www.mdpi.com/article/10.3390/jcdd8040044/s1, Table S1: Summarized baseline clinical and echocardiographic characteristics grouped by 5-year mortality after TAVI, Table S2: Removal of quasi-constant/constant features (features that have approximately 99% of the values are similar) and correlated features (threshold 0.7), Table S3: Hyperparameters for random forest, gradient boosting, and multilayer perceptron algorithms.
