# Supplementary material for: Predicting Long-Term Mortality in TAVI Patients Using Machine Learning Techniques
Source: J Cardiovasc Dev Dis. 2021 Apr 16;8(4):44. doi: 10.3390/jcdd8040044 (PMC8072967; doi:10.3390/jcdd8040044)
Supplement: Supplementary file 1 [file jcdd-08-00044-s001.zip › jcdd-1118933-supplementary.pdf]

Table S1 – Summarized baseline clinical and echocardiographic characteristics grouped by 5-years mortality after TAVI.

	**Survivor (n = 259)**	**Non-Survivor (n = 212)**	*p*-value
Age (year)	80 ± 6	82 ± 6	0.025
Men	95 (36.7%)	76 (35.8%)	0.852
Body surface area (m^2)	1.71 ± 0.20	1.69 ± 0.18	0.234
Body mass index (Kg/m^2)	26 ± 5	25 ± 5	0.276
Sinus rhythm	225 (86.9%)	152 (71.7%)	<0.001
Diabetes mellitus II	61 (23.6%)	61 (28.8%)	0.198
Diabetes on insulin therapy	15 (5.8%)	16 (7.5%)	0.445
Hypertension	230 (88.8%)	181 (85.4%)	0.267
Dyslipidemia	161 (62.2%)	115 (54.2%)	0.083
Angina	90 (34.7%)	57 (26.9%)	0.057
Dyspnea	234 (90.3%)	198 (93.4%)	0.232
Syncope	54 (20.1%)	33 (15.6%)	0.142
COPD	65 (25.1%)	66 (31.1%)	0.146
Chronic lung disease	66 (25.5%)	51 (24.1%)	0.722
NYHA functional class III or IV	191 (73.7%)	178 (84.0%)	0.007
EuroSCORE II	14 (8-20)	18 (12-25)	<0.001
Previous stroke	29 (11.2%)	31 (14.6%)	0.267
Porcelain aorta	21 (8.1%)	11 (5.2%)	0.210
Peripheral vascular disease	106 (40.9%)	95 (44.8%)	0.396
Coronary artery disease	140 (54.1%)	130 (61.3%)	0.113
Number vessel disease	1 (0–2)	1 (0–2)	0.279
Previous CABG	37 (14.3%)	34 (16.0%)	0.597
Previous PCI	82 (31.7%)	62 (29.2%)	0.571
Prior myocardial infarction	47 (18.1%)	45 (21.2%)	0.402
Recent myocardial infarction (<90 days)	6 (2.3%)	6 (2.8%)	0.725
Atrial fibrillation	30 (11.6%)	56 (26.4%)	<0.001
AV block pacemaker	20 (7.7%)	15 (7.1%)	0.790
Paced at baseline	17 (6.6%)	33 (15.6%)	0.002
Poor mobility	63 (24.3%)	63 (29.7%)	0.188
Neurological dysfunction	30 (11.6%)	17 (8.0%)	0.199
Frailty	21 (8.1%)	52 (24.5%)	<0.001
Cognitive dysfunction/dementia	25 (9.7%)	26 (12.3%)	0.364
Critical preoperative state	7 (2.7%)	10 (4.7%)	0.244
Previous cardiac surgery	44 (17.0%)	46 (21.7%)	0.196
Ascites	1 (0.4%)	3 (1.4%)	0.226
Cirrhosis	6 (2.3%)	10 (4.7%)	0.153
Dialysis	1 (0.4%)	3 (1.4%)	0.226
Hostile chest	54 (20.8%)	55 (25.9%)	0.192
Previous neoplasm	59 (22.8%)	48 (22.6%)	0.972
Active endocarditis	0 (0%)	0 (0%)	/
Procedural urgency	12 (4.6%)	9 (4.2%)	0.839
Creatinine (mg/dl)	0.92 (0.77–1.20)	1.16 (0.91–1.48)	<0.001
Hemoglobin (g/dl)	12.4 ± 1.7	11.9 ± 1.6	<0.001
C reactive protein ≥6 (mg/dl)	63 (24.3%)	76 (35.8%)	0.006
Total bilirubin (μmol/l)	0.73 (0.59–0-95)	0.78 (0.59–1.06)	0.225
Aspartate transaminase (U/l)	20 (17–27)	21 (17–28)	0.573
Alanine aminotransferase (U/l)	17 (12–23)	16 (12–22)	0.448
International normalized ratio	1.05 ± 0.19	1.17 ± 0.42	<0.001
B-blocker	122 (47.1%)	87 (41.0%)	0.187

Diuretics	177 (68.3%)	175 (82.5%)	<0.001
Spironolactone	42 (16.2%)	63 (29.7%)	<0.001
Calcium channel blocker	60 (23.2%)	50 (23.6%)	0.915
Statins	113 (43.6%)	78 (36.8%)	0.133
Anticoagulant	61 (23.6%)	38 (17.9%)	0.136
Angiotensin converting enzyme inhibitor and/or angiotensin II receptor blocker	156 (60.2%)	123 (58.0%)	0.627
Systolic blood pressure (mmHg)	128 ± 16	126 ± 17	0.085
Diastolic blood pressure (mmHg)	72 ± 9	70 ± 10	0.141
LVEDD (mm)	48 ± 8	49 ± 8	0.220
IVST (mm)	13 ± 2	14 ± 2	0.496
Posterior wall thickness (mm)	12 ± 2	12 ± 2	0.841
LV mass index (g/m^2)	143 ± 39	151 ± 40	0.036
LVEDV index (ml/m^2)	52 (42–66)	55 (43–70)	0.118
LVESV index (ml/m^2)	20 (15–31)	23 (16–37)	0.014
LVEF (%)	60 (52–66)	58 (43–64)	0.003
Left atrium area (cm^2)	26 ± 6	28 ± 7	<0.001
Left atrial volume index (ml/m^2)	53 ± 25	61 ± 22	0.001
LVOT (mm)	20 ± 2	20 ± 2	0.853
Aortic annulus (mm)	21 ± 2	21 ± 2	0.646
Ascending aorta (mm)	34 ± 4	33 ± 4	0.369
Grade aortic valve calcium	3.0 ± 0.4	3.0 ± 0.5	0.662
Mean aortic pressure gradient (mmHg)	53 ± 14	48 ± 15	0.001
Peak aortic pressure gradient (mmHg)	85 ± 21	79 ± 23	0.002
LVOT_VTI (cm)	22 ± 4	21 ± 5	0.003
LV stroke volume (ml/m^2)	42 ± 8	40 ± 9	0.020
Aortic valve area (cm^2)	0.64 ± 0.14	0.66 ± 0.14	0.078
Aortic regurgitation ≥2	69 (26.6%)	51 (24.1%)	0.522
Mitral regurgitation ≥2	65 (25.1%)	79 (37.3%)	0.004
Tricuspid regurgitation ≥2	37 (14.3%)	52 (24.5%)	0.005
Mitral regurgitation etiology			<0.001
Function	182 (70.3%)	113 (53.3%)	
Organic	77 (29.7%)	99 (46.7%)	
Previous mitral valve repair	4 (1.5%)	0 (0%)	0.131
Previous mitral valve replacement	2 (0.8%)	10 (4.7%)	0.008
Systolic pulmonary artery pressure (mmHg)	40 ± 11	44 ± 12	<0.001
Prosthesis to indexed annulus size ratio	2.38 ± 0.46	2.37 ± 0.51	0.815

Values are mean ± SD, median (twenty-fifth–seventy-fifth percentile) or n(%).
AV, atrioventricular; CABG, coronary artery bypass graft; COPD, chronic obstructive pulmonary disease; EDD, end diastolic diameter; EDV, end diastolic volume; EF, ejection fraction; ESV, end systolic volume; IVST, interventricular septal thickness; LV, left ventricular; LVOT, left ventricular outflow tract; NYHA, NewYork Heart Association; PCI, percutaneous coronary intervention; VTI, velocity time integral. *: $p < 0.05$, survivors vs non-survivors (unpaired Student's t test, Mann-Whitney U test, or $\chi 2$ test).

Table S2 – Remove quasi-constant/constant features (features that have approximately 99% of the values are similar) and correlated features (threshold 0.7).

Constant features	Correlated features
Previous mitral valve repair	Aspartate transaminase
Active endocarditis	Sinus rhythm
Dialysis	Left atrial volume index
Ascites	LVESV index
	CABG
	Peak aortic pressure gradient
	LVOT_VTI
	Number vessel disease
	LVEDV index

CABG, coronary artery bypass graft; EDV, end diastolic volume; ESV, end systolic volume; LV, left ventricular; LVOT, left ventricular outflow tract; VTI, velocity time integral.

Table S3 – Hyperparameters for random forest, gradient boosting and multilayer perceptron algorithms.

Hyperparameter tuning of the models was conducted by grid search using five-fold cross-validation. The scoring function used was F1-score. The hyper-parameter space searched over are presented below.

Random Forest	
n_estimators	[50,60,80,100,200,400,500]
max_depth	[3–7]
min_sample_split	[3–8]
min_samples_leaf	[1–4]
**Gradient boosting machine**	
learning_rate	[0.1,0.01]
n_estimators	[50,60,80,100,200,400,500]
max_depth	[3–7]
min_child_weight	[1–4]
gamma	[0, 0.5,1,1.5,2,5]
colsample_bytree	[0.6,0.8,1]
**Multilayer perceptron**	
batch size	[1,2,4,8]
hidden layer	[1–3]
learning rate	[0.1,0.01]
dropout rate	[0.0,0.2,0.3,0.5]
dense layer units	[5,10,15]
weight initialization	['uniform', 'zero', 'he_normal', 'he_uniform']

Multilayer perceptron was implemented in keras. Optimizer='Adam', dense layer activation function='Relu', output layer activation function="sigmoid", loss function="binary_crossentropy", epochs=100 with early stopping if the validation loss did not continue to decrease in 10 epochs.

```
import tensorflow as tf
from tensorflow import keras
from tensorflow.keras import layers
from tensorflow.keras import backend as K
from tensorflow.keras.models import Sequential
from tensorflow.keras.layers import Dense, Activation
from tensorflow.keras.layers import Dropout
from tensorflow.keras import regularizers
from tensorflow.keras import initializers
from tensorflow.keras.callbacks import EarlyStopping

def create_nn(input_shape,
              hidden_layers=1,
              dropout_rate = 0.0,
              learn_rate = 0.01,
              neurons = 5,
              activation = 'relu',
              optimizer = 'adam',
              init_mode = 'uniform'):
    model = Sequential()

    model.add(Dense(15,activation=activation,
                    input_shape=input_shape,
                    kernel_initializer=init_mode))
    model.add(Dropout(dropout_rate))

    for i in range(hidden_layers):
        model.add(Dense(neurons, activation=activation,
kernel_initializer=init_mode))
        model.add(Dropout(dropout_rate))

    model.add(Dense(1, activation='sigmoid', kernel_initializer=init_mode))

    Optimizer = optimizer(lr=learn_rate)
    model.compile(
        loss='binary_crossentropy',
        optimizer=Optimizer,
        metrics=['accuracy']
        #metrics=['accuracy', f1_m, precision_m, recall_m]
    )  #mean_absolute_error  #binary_crossentropy

    return model

from tensorflow.keras import backend as K

def recall_m(y_true, y_pred):
  true_positives = K.sum(K.round(K.clip(y_true * y_pred, 0, 1)))
  possible_positives = K.sum(K.round(K.clip(y_true, 0, 1)))
```

```
  recall = true_positives / (possible_positives + K.epsilon())
  return recall

def precision_m(y_true, y_pred):
  true_positives = K.sum(K.round(K.clip(y_true * y_pred, 0, 1)))
  predicted_positives = K.sum(K.round(K.clip(y_pred, 0, 1)))
  precision = true_positives / (predicted_positives + K.epsilon())
  return precision

def f1_m(y_true, y_pred):
  precision = precision_m(y_true, y_pred)
  recall = recall_m(y_true, y_pred)
  return 2*((precision*recall)/(precision+recall+K.epsilon()))


param_grid = {'batch_size': [1,2,4,8],
              #'optimizer' : ['Adam', 'Nadam'],
              'dropout_rate' : [0.0, 0.2, 0.3, 0.5],
              'learn_rate' : [0.1, 0.01],
              'neurons' : [5,10,15],
              'hidden_layers ' : [5,10,15],
              'init_mode' : ['uniform', 'zero', 'he_normal', 'he_uniform'],
              #'activation' : ['relu', 'elu']
             }


model = KerasClassifier(build_fn = create_nn, input_shape=(shape,), epochs=100,
verbose=0)

# Fit the model
gs = GridSearchCV(
    estimator=model,
    param_grid=param_grid,
    cv=cv,
    n_jobs=-1,
    verbose=2
)
hist = gs.fit(Xtrain, Ytrain)
print("Best: %f using %s" % (hist.best_score_, hist.best_params_))

fitted_model = hist.best_estimator_

scores  = fitted_model.evaluate(x_test, y_test, verbose=0)

print('%s: %.2f' % (fitted_model.metrics_names[0], scores[0]))
cvloss.append(scores[0])
yhat_classes = fitted_model.predict_classes(x_test, verbose=0)
yhat_probs = fitted_model.predict(x_test, verbose=0)
yhat_classes = yhat_classes[:, 0]
yhat_probs = yhat_probs[:, 0]
```
